# Predicting resting energy expenditure in underweight, normal weight, overweight, and obese adult hospital patients

**DOI:** 10.1186/s12986-016-0145-3

**Published:** 2016-11-24

**Authors:** Hinke M. Kruizenga, Geesje H. Hofsteenge, Peter J.M. Weijs

**Affiliations:** 1 Department of Nutrition and Dietetics, Internal Medicine, VU University Medical Center, P.O. Box 7057, Amsterdam, 1007 MB The Netherlands; 2 Department of Nutrition and Dietetics, School of Sports and Nutrition, Amsterdam University of Applied Sciences, Amsterdam, The Netherlands

**Keywords:** Resting energy expenditure, Equation, BMI, Prediction, Validity, Underweight, Normal weight, Overweight, Obese, Indirect calorimetry

## Abstract

**Background:**

When indirect calorimetry is not available, predictive equations are used to estimate resing energy expenditure (REE). There is no consensus about which equation to use in hospitalized patients. The objective of this study is to examine the validity of REE predictive equations for underweight, normal weight, overweight, and obese inpatients and outpatients by comparison with indirect calorimetry.

**Methods:**

Equations were included when based on weight, height, age, and/or gender. REE was measured with indirect calorimetry. A prediction between 90 and 110% of the measured REE was considered accurate. The bias and root-mean-square error (RMSE) were used to evaluate how well the equations fitted the REE measurement. Subgroup analysis was performed for BMI. A new equation was developed based on regression analysis and tested.

**Results:**

513 general hospital patients were included, (253 F, 260 M), 237 inpatients and 276 outpatients. Fifteen predictive equations were used. The most used fixed factors (25 kcal/kg/day, 30 kcal/kg/day and 2000 kcal for female and 2500 kcal for male) were added. The percentage of accurate predicted REE was low in all equations, ranging from 8 to 49%. Overall the new equation performed equal to the best performing Korth equation and slightly better than the well-known WHO equation based on weight and height (49% vs 45% accurate). Categorized by BMI subgroups, the new equation, Korth and the WHO equation based on weight and height performed best in all categories except from the obese subgroup. The original Harris and Benedict (HB) equation was best for obese patients.

**Conclusions:**

REE predictive equations are only accurate in about half the patients. The WHO equation is advised up to BMI 30, and HB equation is advised for obese (over BMI 30). Measuring REE with indirect calorimetry is preferred, and should be used when available and feasible in order to optimize nutritional support in hospital inpatients and outpatients with different degrees of malnutrition.

## Background

In clinical practice, an adequate measurement of resting energy expenditure (REE) for adult patients is important for optimal nutritional therapy in order to prevent under- and over nutrition [1]. REE in adult patients can be measured by indirect calorimetry, based on oxygen consumption and carbon dioxide production [2]. Indirect calorimetry is considered as the most accurate method [3] for determining the REE in adult patients [4, 5]; however, this measurement is time-consuming and not available in most clinical settings. As an alternative, REE is usually calculated with various REE predictive equations, based on healthy subjects [1, 6].

Only few studies have validated REE predictive equations in hospitalized patients [7–9]. The number of validated predictive equations is small [7, 8] and studies have small sample sizes [7, 9]. Therefore, there is no consensus about which equation to use in hospitalized patients. According to Boullata et al. [8], the Harris & Benedict (1918) (HB1918) [10] equation is the best equation to predict REE, when using an illness factor of 1.1. It appeared 62% of the patients were predicted accurately using this equation. Anderegg et al. [7] suggests HB1918 with adjusted bodyweight and a stress factor, which led to 50% accurately predicted patients. Weijs et al. [9] suggest the WHO and adjusted Harris & Benedict (HB1984) [11] equations, predicting about 50% of the patients accurately. More recently, Jesus et al. [12] showed that the original Harris & Benedict equation (HB1918) performed reasonably, but no equation was adequate for extreme BMI groups (<16 and >40).

Therefore, it is unclear which REE predictive equation performs most uniform across BMI subgroups for hospital patients. The aim of this study is to examine the validity of REE predictive equations for underweight, normal weight, overweight, and obese patients by comparison with indirect calorimetry.

## Methods

### Patients

Between March 2005 and December 2015, data were collected at the VU University Medical Center Amsterdam. Patients who had an indication for nutritional assessment by the dietitian were included in this study. All measurements were performed according to a standardized operating procedure (SOP), and personal was trained in a standardized manner. Patients were measured as part of patient care. As malnutrition is the main reason for measurement, withholding food for longer than absolutely necessary is questionable and maybe unethical. All patients were restricted from food for at least 2 h before the measurement. None of the patients were restricted from food for 8 h, as the guideline [13] indicates.

Only adult patients with complete data (height, weight, age, and gender) were included. When repeated REE measurements were available, only the first measurement was included. Exclusion criteria were patients at ICU, pregnant women, and REE measurements shorter than 15 min. All procedures were in accordance with ethical standards of the institution.

### Indirect calorimetry and anthropometric measurements

Indirect calorimetry measurements were performed by using a metabolic monitor (Deltatrac 2 MBM-200, Datex-Ohmeda, Helsinki, Finland; Vmax Encore n29, Viasys Healthcare, Houten, The Netherlands). Both devices were calibrated every day before use and Vmax also every 5 min during measurement. The Deltatrac was calibrated with one reference gas mixture (95% O_2_, 5% CO_2_), whereas Vmax was calibrated with two standard gases (26% O_2_, 0% CO_2,_ and 16% O_2_, 4% CO_2_). Patients were measured in supine position. Calibration and measurements were performed by a trained dietitian. Oxygen analyser sensitivity was checked yearly by supplier.

Body weight was measured using a calibrated electronic stand-up scale (Seca Alpha, Hamburg, Germany). In case of severe oedema or when weighing was not possible, even weighing in bed, self-reported weight was used. Height of the patient was measured or self-reported. BMI was calculated as weight (kg) divided by the square of height (m^2^).

### REE predictive equations

Predictive equations were obtained by a systematic search using PubMed. Mesh-derived keys ‘energy metabolism’, ‘basal metabolism’ and ‘indirect calorimetry’ and additional terms (‘predict*’, ‘estimat*’, ‘equation*’ and ‘formula*’) were applied in every possible combination. Applied limitations were ‘English language’, ‘humans’ and the age of 18 years and older. Additional publications were checked based on reference lists. Equations were included when based on body weight, height, age, and/or gender.

The Weijs equation for overweight patients [14] was tested in patients with BMI > 25. For the BMI < 25 subgroup, a new REE predictive equation was developed in this subpopulation with BMI < 25 using regression analysis with measured REE (kcal/day) as dependent and body weight (kg), height (m), age (y), and sex (F = 0, M = 1) as independent variables.

### Statistical analysis

An independent samples *T*-test was used for differences in weight, BMI, age, and REE between inpatients and outpatients, as well as between males and females. BMI subgroups were analysed: underweight (BMI < 18.5 kg/m^2^), normal weight (BMI ≥18.5- < 25 kg/m^2^), overweight (BMI ≥25- < 30 kg/m^2^), and obese patients (BMI ≥ 30 kg/m^2^). The difference between the REE predictive equation and REE measured was calculated as percentage. A prediction between 90 and 110% of the REE measured was considered as accurate prediction. A prediction below 90% was considered as under prediction and a prediction over 110% was considered as over prediction. The bias indicates the mean percentage error between REE predictive equation and REE measured. The root-mean-square-error (RMSE), expressed in kcal/day, was used to measure how well the equations fitted the REE measurement.

To check whether in underweight and obese patients adjustment of weight in the REE predictive equation resulted in a better performance of the equation, body weight adjustment was applied (BMI < 18.5: weight adjusted to BMI = 18.5); BMI > 30: weight adjusted to BMI = 30). The criterion for improvement of performance was percentage accurate predictions. Statistical significance was reached when *p* < 0.05. Data was analysed with IBM SPSS Statistics 20.

## Results

### Patients

Table 1 shows characteristics of study populations. REE measurements of 593 patients were available. Eighty had incomplete data. In total, 513 general hospital patients were included, (253 F, 260 M), 237 inpatients and 276 outpatients. These patients were often complex patients with multimobidity and were categorised as oncology (29%), gastroenterology (19%, Diabetes/overweight (14%), Nephrology (10%), Lung diseases (7%), Neurology (5%), diagnostics in unintentional weight loss (5%) and a rest group (8%) of cardiology, anorexia nervosa, auto immune disease, spinal cord injury and RA patients.

**Table 1:** Patient characteristics for the total group and per BMI group

	Total group		BMI < 18,5		BMI 18,5–25		BMI 25–30		BMI > 30	
N (%)	513		141 (27%)		209 (41%)		77 (15%)		86 (17%)	
	Mean	SD	Mean	SD	Mean	SD	Mean	SD	Mean	SD
Age (y)	53.0	15.6	51.3	17.0	54.1	15.2	55.3	15.2	50.9	14.2
% Male	51%		44%		58%		53%		41%	
Weight (kg)	70.1	22.9	49.4	7.3	64.2	8.7	83.2	11.0	106.7	21.3
Height (m)	1.73	0.10	1.72	0.10	1.74	0.09	1.74	0.10	1.71	0.12
BMI (kg/m^2^)	23.4	7.2	16.6	1.5	21.3	1.8	27.3	1.4	36.3	5.4
REE (kcal/day)	1678	408	1448	318	1696	358	1730	352	1966	488
REE in kcal/kg/day (range)	25.1 (12–53)	6.2	29.4 (18–43)	5.5	26.6 (14–53)	5.3	20.8 (12–31)	3.3	18.5 (13–29)	3.2
% inpatients	46%		57%		55%		35%		17%	

### REE predictive equations

In total, 15 predictive equations were used. The most used fixed factors (25 kcal/kg/day, 30 kcal/kg/day and 2000 kcal for female and 2500 kcal for male) were added. These fixed factors calculate total energy expenditure and in order to provide REE, they were divided by a physical activity and/or stress factor of 1.3. Appendix 1 shows the descriptives of the included REE predictive equations.

### Accuracy of predictive equations

Based on REE data of patients with BMI < 25 a new equation was developed in the current population: BMI < 25: REE (kcal/day) = 11.355 × weight (kg) + 7.224 × height (cm) - 4.649 × age (y) + 135.265 × sex (F = 0; M = 1) - 137.475; for BMI ≥ 25 an equation had been developed on healthy overweight and/or obese subjects by Weijs and Vansant [14]: BMI ≥ 25: REE (kcal/day) = 14.038 × weight (kg) + 4.498 × height (cm) - 0.977 × age (y) + 137.566 × sex (F = 0; M = 1) - 221.631.

Table 2 shows statistics of the REE predictive equations for all patients. The percentage of accurate predicted REE was low in all equations, ranging from 8 to 49%. Overall the new equation performed equal to the best performing Korth equation and slightly better than the well-known WHO equation based on weight and height (49% vs 45% accurate).

**Table 2:** Statistics of REE prediction equation performance, N = 513

	REE (kcal/day)	SD	Under prediction (%) ^a^	Accurate prediction (%) ^b^	Over prediction (%) ^c^	BIAS ^d^	RMSE ^e^
REE by calorimetrie	1678	408					
New equation	1698	313	19	49	32	4	286
Korth [18]	1621	344	30	49	22	−1	295
WHO-wtht [15]	1540	288	40	45	14	−6	321
Schofield-wtht [19]	1513	282	46	42	12	−7	333
Henry-wtht [20]	1489	291	51	39	10	−9	344
WHO-wt [15]	1504	304	49	39	13	−8	345
Harris& Benedict 1918 [10]	1490	324	51	38	11	−9	350
Muller [21]	1493	308	52	37	11	−9	347
H&B by Roza [11]	1494	321	53	37	11	−9	344
Schofield-wt [19]	1483	293	53	36	12	−9	355
Mifflin [22]	1444	304	60	32	8	−12	369
Henry2005-wt [20]	1458	320	58	31	10	−11	370
MullerBMI [21]	1396	435	60	31	9	−16	450
30 kcal/kg	1618	527	44	28	28	−2	435
Livingston [23]	1405	284	66	27	7	−14	399
25 kcal/kg	1348	440	68	23	9	−19	502
2000 kcal for female and 2500 kcal for male	2253	250	3	11	87	41	689
Bernstein [24]	1208	271	90	8	2	−26	557

^a^ The percentage of subjects predicted by this predictive equation < 10% of the measured value

^b^ The percentage of subjects predicted by this predictive equation within 10% of the measured value

^c^ The percentage of subjects predicted by this predictive equation > 10% of the measured value

^d^ Mean percentage error between predictive equation and measured value

^e^ Root mean squared prediction error

Table 3 shows statistics for the best predictive equations categorized by BMI subgroups. The new equation, Korth and the WHO equation based on weight and height performed best in all categories except from the obese subgroup. HB1918 was best for obese patients.

**Table 3:** REE predictive accuracy of prediction equations in BMI subgroups

	Total group (n = 513)			BMI <18.5 (n = 141)			BMI 18.5–25 (n = 209)			BMI 25–30 (n = 77)			BMI > 30 (n = 86)		
	Under predic-tion	Accu-rate	Over predic-tion	Under predic-tion	Accu-rate	Over predic-tion	Under predic-tion	Accu-rate	Over predic-tion	Under predic-tion	Accu-rate	Over predic-tion	Under predic-tion	Accu-rate	Over predic-tion
	%	%	%	%	%	%	%	%	%	%	%	%	%	%	%
New equation	19	49	32	21	44	35	22	51	27	14	58	27	14	44	42
Korth [18]	30	49	22	35	40	24	34	52	14	17	56	27	22	48	30
WHO-wtht [15]	40	45	14	40	45	14	48	43	9	27	55	18	33	44	23
Schofield-wtht [19]	46	42	12	43	44	13	54	40	7	36	48	16	40	40	21
Henry-wtht [20]	51	39	10	50	37	13	60	35	4	40	45	14	38	45	16
WHO-wt [15]	49	39	13	52	35	12	60	33	7	30	53	17	31	45	23
Harris & Benedict 1918 [10]	51	38	11	60	27	13	63	33	3	34	53	13	26	53	21
Muller [21]	52	37	11	59	29	12	62	33	5	38	48	14	28	51	21
H&B by Roza [11]	53	37	11	57	30	13	65	33	3	39	45	16	29	50	21
Schofield-wt [19]	53	36	12	54	33	13	61	32	7	42	47	12	40	40	21
Mifflin [22]	58	33	8	60	28	12	66	30	4	45	45	9	48	40	13
Henry-wt [20]	60	32	8	60	28	12	67	29	4	49	43	8	52	37	10
MullerBMI [21]	60	32	8	63	27	10	70	27	3	48	43	9	43	43	14
30 kcal/kg	58	31	10	67	23	11	69	27	4	40	45	14	35	43	22
Livingston [23]	60	31	9	99	1	0	55	36	9	39	47	14	29	50	21
25 kcal/kg	44	28	28	78	16	6	51	39	11	8	40	52	5	10	85
2000 kcal for female and 2500 kcal for male	66	27	7	73	19	8	73	23	3	53	39	8	50	38	12
Bernstein [24]	68	23	9	91	9	0	86	12	2	40	49	10	14	47	40

Accurate prediction: the percentage of subjects predicted by this predictive equation within 10% of the measured value

Underprediction: the percentage of subjects predicted by this predictive equation <10% of the measured value

Overprediction: the percentage of subjects predicted by this predictive equation > 10% of the measured value

**Fig. 1:**
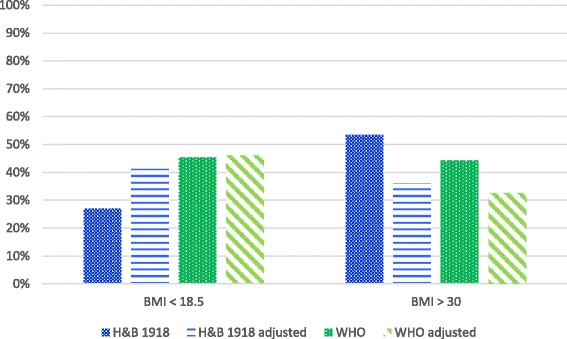
The percentage of accurately predicted underweight and obese patients with actual as well as adjusted weight (BMI < 18.5: weight adjusted to BMI = 18.5); BMI > 30: weight adjusted to BMI = 30)

Figure 1 shows the percentage of accurately predicted underweight and obese patients with actual as well as adjusted weight using the WHO equation with weight and height [15] and HB1918 [10]. Adjusting the weight in the equation in underweight and obese patient did not improve the percentage of patients with an accurate predicted REE.

## Discussion

This study shows that for hospital inpatients and outpatients the generally applied WHO [15] and the original Harris & Benedict equation (HB1918) [10] can only predict resting energy expenditure accurately in one of two to three patients. The generally used fixed 25 kcal/kg body weight was only accurate in 28% of the patients. The Korth equation also performed well, but not significantly better than the well implemented WHO and H&B equations. The newly developed equation performed equal to the best performing equations but showed no additional value. Generally applied weight adjustments all failed to improve accuracy. Hospital inpatients and outpatients may still benefit from using indirect calorimetry for assessment of energy needs.

Studies by Anderegg et al. [7] and Boullata et al. [8] analysed (in part) mechanically ventilated patients and are therefore more difficult to compare to current inpatient and outpatient analysis. However, in general they also showed rather inaccurate estimates using different REE estimating equations. Based on a similar analysis with a much smaller sample size, Weijs et al. [9] concluded that the WHO equation (1985) [15] based on weight and height and Harris & Benedict (1984) [11] were the best predictive equations. The current analysis confirms that the overall accuracy of REE predictive equations is only about 50%, however this study extends this analysis to BMI subgroups for which predictive accuracy may in fact be much worse.

Jesus et al. [12] showed that the overall accuracy of the Harris & Benedict equation was reasonable for the outpatient sample. The authors stress that predictive accuracy is much worse in extreme BMI subgroups with BMI under 16 and BMI over 40. The current study generally supports these conclusions, however extend these observations in two ways. First, the general accuracy is not that much higher in the normal weight patient group, in fact accuracy increases to highest level in overweight subgroup. Secondly, we agree that the subgroup of patients with BMI less than 16 has a low prediction accuracy, however we have also shown low prediction accuracy for a large cohort of malnourished hospitalized elderly with mean BMI 21 (SD 4) [16]. Therefore, the suggestion that predictive equations perform well between BMI 16 and BMI 40 is largely false for hospital patients.

According to Frankenfield et al. [17], adjusting body weight in obese patients leads to underestimation of the energy expenditure. When this is done with a fixed BMI level, the adjustment appears too large and does not result in a higher accuracy of REE prediction. However, accuracy remains low in all predictions.

This study has several strengths and limitations. The sample size of 513 patients was large enough for subgroup analysis, namely BMI subgroups. Furthermore, these data were derived from daily clinical practice and therefore the study population is representative for the inpatient and outpatient population. Another advantage is the exclusion of ICU patients that may not be entirely comparable to the general hospital population. Therefore, this study has a large generalizability to other hospitals and patients.

However, this study has some limitations as well. The measurements were performed in clinical practice and therefore patients were not measured in overnight fasted state. However, since patients were measured because of nutritional problems, the thermic effect of larger meals, if any, were not a problem in this patient sample. This could have been a problem in obese outpatients, however according to the results the estimations are most accurate in this subgroup. Only when the dietitian indicated the patient for nutritional assessment, a measurement was performed. This may have led to selection bias as only patients who were difficult to assess and/or treat were included in this study. This may largely explain the low level of accuracy in the current analysis.

This study population was too small to develop a new equation for the hospital in and outpatients. The variation of REE between patients and probably between disease groups is too large. A possible way forward, is to develop new equations in more homogenous subgroups. For this purpose a very large database would be needed on REE in hospital patients. We propose to develop an REE repository for clinical data, comparable to the Oxford database on REE in healthy subjects. This could be jointly organised within ESPEN and ASPEN.

## Conclusions

In conclusion, REE predictive equations are only accurate in about half the patients. The WHO equation is advised up to BMI 30, and HB1918 equation is advised for obese (over BMI 30). Measuring REE with indirect calorimetry is preferred, and should be used when available and feasible in order to optimize nutritional support in hospital inpatients and outpatients with different degrees of malnutrition.

## Appendix 1

**Table 4:** Descriptives of included predictive equations

Author, year of publication and referred to as	Study population and n	Age (mean ± SD or range)	REE Equations (kcal/day)
Bernstein, 1983 [24]	Obese individuals; patients who enrolled the Weight Control Unit of the Obesity Research Center IC instrument: Beckman *n*: 48 M/154 F	M: 39 ± 12 y	M: 11.02 × WT + 10.23 × HTCM - 5.8 × AGE – 1032
		F: 40 ± 13 y	F: 7.48 × WT - 0.42 × HTCM - 3.0 × AGE + 844
FAO/WHO/UNU, 1985 [15] WHO-wt WHO-wtht	*n*: 575 M/734 F	All: 30–82y	M 18–30: (15.3 × WT) + 679
			F 18–30: (14.7 × WT) + 496
			M 30–60: (11.6 × WT) + 879
			F 30–60: (8.7 × WT) + 829
			M 60+: (13.5 × WT) + 487
			F 60+: (10.5 × WT) + 596
			Equations based on weight and height
			M 18–30: (15.4 × WT) – (27 × HTM) + 717
			F 18–30: (13.3 × WT) + (334 × HTM) + 35
			M 30–60: (11.3 × WT) + (16 × HTM) + 901
			F 30–60: (8.7 × WT) - (25 × HTM) + 865
			M 60+: (8.8 × WT) + (1128 × HTM) – 1071
			F 60+: (9.2 × WT) + (637 × HTM) – 302
Harris & Benedict, 1918 [10]	*n*: 136 M/103 F	M: 27 ± 9 (16–63) y	M: 66.4730 + (13.7516 × WT) + (5.0033 × HTCM) – (6.7550 × AGE)
		F: 31 ± 14 (15–74) y	F: 655.0955 + (9.5634 × WT) + (1.8496 × HTCM) – (4.6756 × AGE)
Harris & Benedict, 1984 Roza & Shizgal [11] H&B by Roza	Data of Harris & Benedict (1918) and data of two further studies by Benedict with data on additional subjects (*n*: 168 M/169 F)	M: 30 ± 14 y	M: 88.362 + (13.397 × WT) + (4.799 × HTCM) – (5.677 × AGE)
		F: 44 ± 22 y	F: 447.593 + (9.247 × WT) + (3.098 × HTCM) – (4.330 × AGE)
Korth, 2007 [18]	Healthy euthyroid weight stable subjects who were recruited by local announcements *n*: 50 M/54 F	M: 39 ± 14 (21–68) y	All: (41.5 × WT) – (19.1 × AGE) + (35.0 × HTCM) + (1107.4 × SEX) – 1731.2/4.184
		F: 35 ± 15 (20–66) y	
Livingston, 2005 [23]	Institute of Medicine population *n*: 299 M/356 F	M: 36 ± 15 (18–95) y	M: 293 × WT ^0.4330^ – (5.92 × AGE)
		F: 39 ± 13 (18–77) y	F: 248 × WT ^0.4356^ – (5.09 × AGE)
Mifflin, 1990 [22]	IC instrument: metabolic measurement cart with a canopy hood (Metabolic Measurement Cart Horizons System) *n*: 251 M (122 obese)/247 F (112 obese)	M: 44 ± 14 (19–78) y	M: (9.99 × WT) + (6.25 × HTCM) – (4.92 × AGE) + 5
		F: 45 ± 14 (20–76) y	F: (9.99 × WT) + (6.25 × HTCM) – (4.92 × AGE) – 161
Muller, 2004 [21]	Data from seven different research centers in Germany IC instruments: Deltatrac, Beckman, Mouthpiece (metabolic chamber) *n* BMI < 18.5: 58 *n* BMI 18.5–25: 444 *n* BMI 25–30: 266 *n* BMI > 30: 278	BMI ≤ 18.5: 32 ± 12 y	All: (0.047 × WT) + (1.009 × SEX) – (0.01452 × AGE) + 3.21/4.184 ×1000
		BMI > 18.5–25: 38 ± 17 y	BMI ≤ 18.5: (0.07122 × WT) – (0.02149 × AGE) + (0.82 × SEX) + 0.731/4.184 ×1000
Muller		BMI > 25–30: 53 ± 16 y	BMI > 18.5–25: (0.02219 × WT) + (0.02118 × HTCM) + (0.884 × SEX) – (0.01191 × AGE) + 1.233/4.184 ×1000
MullerBMI		BMI ≥ 30: 47 ± 13 y	BMI > 25–30: (0.04507 × WT) + (1.006 × SEX) – (0.01553 × AGE) + 3.407/4.184 ×1000
			BMI ≥ 30: (0.05 × WT) + (1.103 × SEX) – (0.01586 × AGE) + 2.924/4.184 ×1000
Henry, 2005 [20]	Worldwide population (excluded Italian subjects) from several papers M 18–30 y: 2821/2816 F 18–30 y: 1664/1655 M 30–60 y: 1010/1006 F 30–60 y: 1023/1023 M 60+ y: 534/533 F 60+ y: 334/324	M 18–30: 22 y	M 18–30 y: (16 × WT) + 545
Henry-wt		F 18–30: 22 y	F 18–30 y: (13.1 × WT) + 558
		M 30–60: 40 y	M 30–60 y: (14.2 × WT) + 593
		F 30–60: 41 y	F 30–60 y: (9.74 × WT) + 694
		M 60+: 70 y	M 60+ y: (13.5 × WT) + 514
		F 60+: 69 y	F 60+ y: (10.1 × WT) + 569
			Equations based on weight and height
Henry-wtht			M 18–30 y: (14.4 × WT) + (313 × HTM) + 113
			F 18–30 y: (10.4 × WT) + (615 × HTM) – 282
			M 30–60 y: (11.4 × WT) + (541 × HTM) – 137
			F 30–60 y: (8.18 × WT) + (502 × HTM) – 11.6
			M 60+ y: (11.4 × WT) + (541 × HTM) – 256
			F 60+ y: (8.52 × WT) + (421 × HTM) + 10.7
Schofield, 1985 [19]	Collection of different authors and papers M 18–30 y: 2879 M 30–60 y: 646 M 60+ y: 50 F 18–30 y: 829 F 30–60 y: 372 F 60+ y: 38	M 18–30: 22 y	M 18–30 y: (0.063 × WT) + 2.896/4.184 × 1000
Schofield-wt		F 18–30: 22 y	F 18–30 y: (0.062 × WT) + 2.036/4.184 × 1000
		M 30–60: 40 y	M 30–60 y: (0.048 × WT) + 3.653/4.184 × 1000
		F 30–60: 40 y	F 30–60 y: (0.034 × WT) + 3.538/4.184 × 1000
		M 60+: 72 y	M 60+ y: (0.049 × WT) + 2.459/4.184 × 1000
		F 60+: 66 y	F 60+ y: (0.038 × WT) + 2.755/4.184 × 1000
			Equations based on weight and height
Schofield-wtht			M 18–30 y: (0.063 × WT) – (0.042 × HTM) + 2.953/4.184 × 1000
			F 18–30 y: (0.057 × WT) + (1.184 × HTM) + 0.411/4.184 × 1000
			M 30–60 y: (0.048 × WT) – (0.011 × HTM) + 3.67/4.184 × 1000
			F 30–60 y: (0.034 × WT) + (0.006 × HTM) + 3.53/4.184 × 1000
			M 60+ y: (0.038 × WT) + (4.068 × HTM) – 3.491/4.184 × 1000
			F 60+ y: (0.033 × WT) + (1.917 × HTM) + 0.074/4.184 × 1000

*M* male, *F* female, *y* years, *WT* weight in kilogram, *HTM* height in meters, *HTCM* height in centimetres; *SEX* (male = 1, female = 0) sex, *REE* resting energy expenditure; *kcal/d* kilocalories a day, *IC* indirect calorimetry

## Abbreviations

BMI: Body Mass Index
H&B: Harris & Benedict
ICU: Intensive Care Unit
REE: Resting energy expenditure
WHO: World Health Organisation
